# The reciprocal regulation between host tissue and immune cells in pancreatic ductal adenocarcinoma: new insights and therapeutic implications

**DOI:** 10.1186/s12943-019-1117-9

**Published:** 2019-12-13

**Authors:** Xiaomeng Liu, Jin Xu, Bo Zhang, Jiang Liu, Chen Liang, Qingcai Meng, Jie Hua, Xianjun Yu, Si Shi

**Affiliations:** 1 Department of Pancreatic Surgery, Fudan University Shanghai Cancer Center, Shanghai, 200032 China; 2 Department of Oncology, Shanghai Medical College, Fudan University, Shanghai, 200032 China; 3 Pancreatic Cancer Institute, Fudan University, Shanghai, 200032 China; 4 Shanghai Pancreatic Cancer Institute, Shanghai, 200032 China

**Keywords:** Pancreatic ductal adenocarcinoma, Immune infiltrate, Stromal cells, Immunotherapy

## Abstract

Pancreatic ductal adenocarcinoma (PDAC) is the fourth leading cause of cancer-related death and is one of the most difficult-to-treat cancers. Surgical resection and adjuvant therapy have limited effects on the overall survival of PDAC patients. PDAC exhibits an immunosuppressive microenvironment, the immune response predicts survival, and activation of immune system has the potential to produce an efficacious PDAC therapy. However, chimeric antigen receptor T (CAR-T) cell immunotherapy and immune checkpoint blockade (ICB), which have produced unprecedented clinical benefits in a variety of different cancers, produce promising results in only some highly selected patients with PDAC. This lack of efficacy may be because existing immunotherapies mainly target the interactions between cancer cells and immune cells. However, PDAC is characterized by an abundant tumor stroma that includes a heterogeneous mixture of immune cells, fibroblasts, endothelial cells, neurons and some molecular events. Immune cells engage in extensive and dynamic crosstalk with stromal components in the tumor tissue in addition to tumor cells, which subsequently impacts tumor suppression or promotion to a large extent. Therefore, exploration of the interactions between the stroma and immune cells may offer new therapeutic opportunities for PDAC. In this review, we discuss how infiltrating immune cells influence PDAC development and explore the contributions of complex components to the immune landscape of tumor tissue. The roles of stromal constituents in immune modulation are emphasized. We also predict potential therapeutic strategies to target signals in the immune network in the abundant stromal microenvironment of PDAC.

## Introduction

Pancreatic ductal adenocarcinoma (PDAC) is the fourth leading cause of cancer-related death in the USA and the seventh leading cause of cancer-related death worldwide, with a 5-year relative survival rate of less than 8% [1, 2]. This dismal prognosis is mostly because PDAC is usually diagnosed at an advanced stage and is resistant to therapy [3]. Even in patients who undergo surgical resection, more than 80% suffer disease relapse. Furthermore, chemotherapy and radiotherapy have not substantially improved the survival of patients over the last several years [4].

The prevention and elimination of cancer cells are dependent on the host’s immune system. Impaired immune effector cell infiltration and inactivation of the immune response contribute to the poor prognosis of PDAC patients. Immunotherapies hold great promise for the future and have produced remarkable recent achievements in different cancers [5]. However, most clinical trials of immune checkpoint blockade (ICB) monotherapies have failed to show activity in PDAC [6]. The combination of gemcitabine with a CD40 agonist, which can promote the accumulation of tumoricidal macrophages, produced a preliminary effect on some selected patients with advanced PDAC [7]. This finding indicates that targeting immune network signals is a promising strategy, but the immunoregulatory mechanisms in PDAC are more complex than expected and need more exploration.

What makes the response of PDAC to immunotherapy different from the responses of other solid tumors is the specific host tissue. PDAC is characterized by an abundant tumor stromal content, where immune cell distribution and function are affected by interactions with other cellular components; these interactions result in the immunosuppressive tumor microenvironment (TME) being relatively complicated [8]. The immunosuppressive TME of PDAC is characterized by T cell exhaustion resulting in the loss of cytotoxic effector functions. The infiltration of multiple types of tumor-promoting immune cells, including myeloid-derived suppressor cells (MDSCs), tumor-associated macrophages (TAMs), regulatory T cells (Tregs) and other immune cells, mediates immune evasion and tumor progression [9]. Some tumor cell-inherent resistance mechanisms, such as the tumor mutational burden and aberrant expression of oncogenic pathways, restrain antitumor immunity [10]. However, the poorly immunogenic nature of PDAC is more likely due to the pronounced desmoplastic microenvironment. The histological hallmark features of PDAC consist of abundant cancer-associated fibroblasts (CAFs), sparse vascular structures, nerve fibers, soluble cellular factors and extracellular matrix (ECM), such as hyaluronan (HA) and collagen [11]. Disrupting the immunosuppressive network and promoting the tumoricidal activity of immune cells might provide new opportunities in the treatment of PDAC [12].

In this review, we explore how infiltrating immune cells influence PDAC development and provide an overview of the principal mechanisms that cellular and other components utilize to impact immune cells in the TME. Considering that PDAC is a desmoplastic tumor associated with immune evasion, we also discuss the immunoregulatory functions of stromal constituents and potential immunotherapeutic targets involved in the interactions between immune cells and host tissue.

### Immune infiltrate contributes to PDAC outcomes

**Fig. 1:**
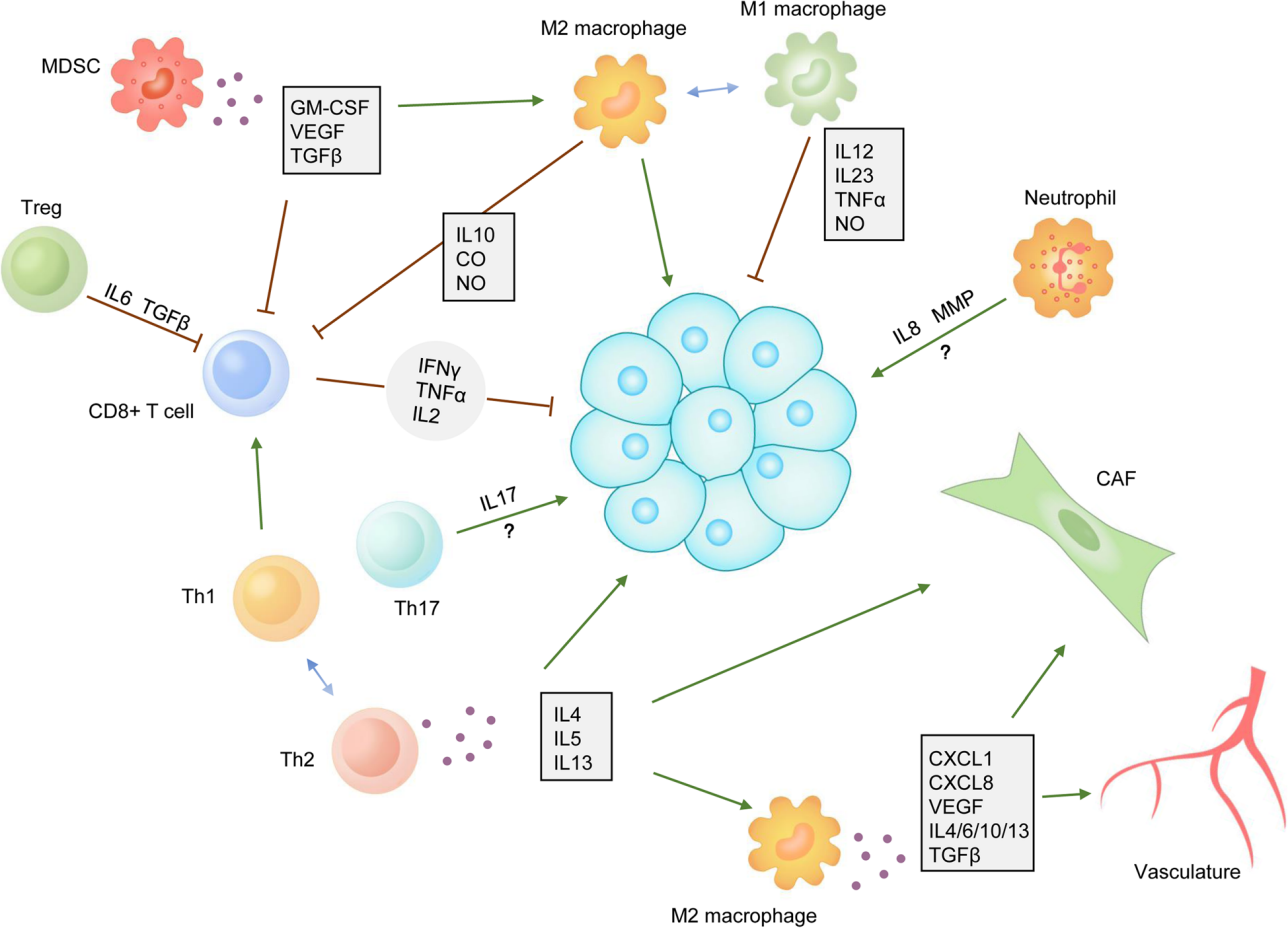
Immune infiltration contributes to PDAC outcomes. PDAC tumor tissue has complex interactions with multiple immune cells, mainly T cells, MDSCs, macrophages and neutrophils. CD8+ T cells eliminate cancer cells by releasing IFNγ and TNFα. CD4+ T cells can be divided into Th1, Th2, and Th17 cells and Tregs. Th1 cells assist CD8+ T cells in antitumor immunity. Th2 cells, which can be reversed into Th1 cells, can drive cancer cell growth and fibroblast activation and can promote the transition of the M1 macrophage into the M2 phenotype. The function of Th17 cells is still not clear, but the functions of these cells mainly depend on IL-17. Tregs inhibit the cytotoxic function of CD8+ T cells via IL-6 and TGFβ. Both MDSCs and M2 macrophages suppress CD8+ T cell functions through the secretion of cytokines. M1 macrophages have antitumor functions that are mediated by releasing IL-12, IL-23, TNFα and NO. M2 macrophages promote tumor progression by secreting cytokines to affect tumor cells, fibroblasts and the vasculature. The role of neutrophils is not clear, but it is known that these cells can exert effects through IL-6 and MMP

The PDAC immune microenvironment is characterized by cytotoxic T lymphocyte (CTL) exhaustion and a strongly suppressive immune cell infiltrate dominated by macrophages [13]. The observed restricted T cell functionality has been shown to be associated with a myeloid-inflamed stroma, which is mediated by myeloid cells such as macrophages, MDSCs and neutrophils [14–16] (Fig. 1).

### T lymphocytes

T lymphocytes represent one of the predominant immune cell subsets and exert both tumor-promoting and tumor-suppressing effects on PDAC. According to their effector functions, mature T cells are classified as CD8+ CTLs and CD4+ helper T (Th) cells, which include Th1, Th2, and Th17 cells and Tregs [17, 18].

With the development of multiplex staining, it has been demonstrated that the proximity of intra-tumoral T cells to PDAC cancer cells is correlated with patient survival. Exclusion of CTLs from the TME represents a tumor escape strategy and results in tumor progression [19–21]. CTLs eliminate tumor cells mainly through IFN-γ-mediated direct effects on malignant cells. However, most CTLs in the PDAC stroma are characterized by upregulated expression of inhibitory receptors, such as T cell immunoglobulin and mucin-domain containing-3, lymphocyte-activation gene-3 and programmed death receptor-1 (PD-1). Signaling through these receptors transforms CTLs into a dysfunctional state named exhaustion, and exhausted T cells have a diminished proliferation ability and lose their cytotoxic functions [22].

Th1 cells may secrete IFN-γ and aid CTLs in tumor rejection, resulting in a positive relationship with prolonged survival [23]. Th2 cells exhibit a tumor-promoting function in PDAC and are related to a poor prognosis [24]. However, the Th2 immune phenotype can be reversed into a preexisting Th1 immune phenotype in a specific context. The ratio of Th1/Th2 tumor-infiltrating lymphocytes predicts survival after surgery in patients with stage IB/III PDAC [25–27]. The tumor-promoting functions of Th2 cells are mediated by the production of cytokines, such as interleukin (IL)-4, IL-5 and IL-13. Th2 cells thereby enhance cancer cell activation, contributing to fibrosis by increasing ECM deposition and collagen synthesis and educating macrophages to differentiate into an M2 immunosuppressive phenotype [28, 29]. Th17 cells exert functions by secreting IL-17, but the influence of Th17 cells on PDAC is paradoxical [23]. Th17 cells have shown an antitumor effect on a mouse model, but studies have also suggested that the IL-17 signaling axis is a potent driver of pancreatic intraepithelial neoplasia [30]. Furthermore, Th17 cells are associated with immune tolerance and diminished survival in PDAC [31]. It is currently unknown what causes Th17 cells have these contradictory effects, but this information may hold the key to the development of successful immunotherapy. The complex crosstalk between T cells and the TME may lead to Th17 infiltration with different impacts on survival.

Tregs are a prominent component of the T lymphocyte population and can be identified with CD4+/CD25+/FOXP3+. Tregs are presumed to exert both pro-tumorigenic and antitumorigenic functions in some tumors. Tregs generally serve as a negative prognostic biomarker and produce suppressive effects on PDAC [32–34]. Tregs can inhibit CTL activation by engaging in extended interactions with tumor-associated CD11c + dendritic cells (DCs), restraining their immunogenic function by suppressing the expression of the costimulatory ligands necessary for CTL activation [35]. Similar to the number of CTLs, the number of CD4+ T cells that secrete IL-17 and IFNγ was shown to increase when Tregs were depleted in PDAC, producing an immunostimulatory environment [36]. The complexity of the immune context is proven by the fact that all T lymphocyte components experience dynamic changes. Dynamic interactions between cancer cells and their microenvironment may contribute to the evolution from immune equilibrium to immune escape.

### Myeloid cells

Myeloid cells have been recognized as important mediators of immune evasion in tumor tissue and are associated with the poor clinical outcome of PDAC. Tumor-associated myeloid cells mainly include macrophages, neutrophils and MDSCs. Recently, these cells have attracted intense interest in PDAC research [37].

Macrophages in PDAC are derived from both inflammatory monocytes and tissue-resident macrophages and play critical roles in the regulation of tumor progression. Based on their distinct functional abilities, they can be categorized into two different states, M1 and M2. The states can change during tumor progression in response to microenvironmental stimuli [38]. M1 macrophages are considered antitumor immune cells that efficiently recognize and destroy cancer cells through phagocytosis and cytotoxicity [39]. These macrophages produce high levels of proinflammatory cytokines, such as IL-12, IL-23, TNFα and chemokines. They also mediate the synthesis of reactive oxygen species (ROS) and the release of nitric oxide (NO) [40]. Increased frequencies of M1 macrophages indicate reduced tumor malignancy, while an elevated M2 macrophage presence suggests decreased survival. TAMs are predominantly considered to exhibit the polarized M2 phenotype in the TME.

TAMs promote tissue repair, immunosuppression and tumor growth by secreting a variety of cytokines, chemokines, and proteases [41, 42]. TAMs regulate vascular structure via the expression of CXCL1, CXCL8 and vascular endothelial growth factor (VEGF). Pharmacological depletion of macrophages in a genetically engineered mouse model (GEMM) of PDAC markedly reduced metastasis formation and was associated with impaired angiogenesis [43, 44]. TAMs are key components of tissue repair that function during chronic wound healing in tumors by releasing profibrotic cytokines [45]. It has been reported that macrophage-secreted granulin supports PDAC metastasis by inducing liver fibrosis. Granulin activates resident hepatic stellate cells, transforming them into myofibroblasts and resulting in a fibrotic microenvironment that sustains metastatic tumor growth [46]. M2 macrophages are widely acknowledged to be an immunosuppressive population within tumors, and M2 macrophage depletion can unleash T cell responses under several therapeutic conditions. iNOS expressed by TAMs can inhibit T cell proliferation according to the potential direct effects of NO on T cells [39]. Interestingly, macrophages could also initiate T cell diapedesis and tumor rejection by generating precisely the amount of NO that promotes endothelial activation [47, 48]. These seemingly contradictory results suggest that the amount of macrophage-derived NO may be valuable for investigation in clinical cancer immunotherapy.

MDSCs are a heterogeneous population that includes immature macrophages, granulocytes and DCs. MDSCs mediate immunosuppression, facilitate tumor progression and correlate with clinical cancer stage. In tumors, MDSCs inhibit proliferation and induce apoptosis in activated T cells. Targeted depletion of granulocytic MDSCs in an autochthonous GEMM of PDAC was shown to increase the intra-tumoral accumulation of CTLs [49]. MDSCs have been shown to exert effects on T cells and other immune cells [50]. They also amplify the immunosuppressive activity of M2 macrophages and DCs via crosstalk and suppress natural killer cell cytotoxicity through cell contact-dependent mechanisms [51]. Moreover, Zhang et al. showed that MDSCs supported immune evasion in PDAC through EGFR/MAPK-dependent regulation of PD-L1 expression on tumor cells [37]. This crosstalk between MDSCs and tumor cells suggests a new way to restore antitumor immunity mediated by CD8+ T cells, a finding with implications for the design of immunotherapies for PDAC.

Neutrophils and polymorphonuclear MDSCs share an origin and many morphological features [52]. Systemic granulocytic expansion has been reported in cancer, correlating with an increased tumor grade and a reduced survival period. Nywening et al. recently showed that targeting neutrophils with small molecule inhibitors augmented antitumor immunity and improved the response to chemotherapy in PDAC [53]. However, the role of neutrophils in pancreatic oncogenesis remains unclear. Recent reports have suggested that neutrophils in tumor tissue can oppose or potentiate cancer progression, which is controlled by signals from cancer cells or stromal cells within the TME [54].

### Immune landscape is shaped by host tissue components

**Fig. 2:**
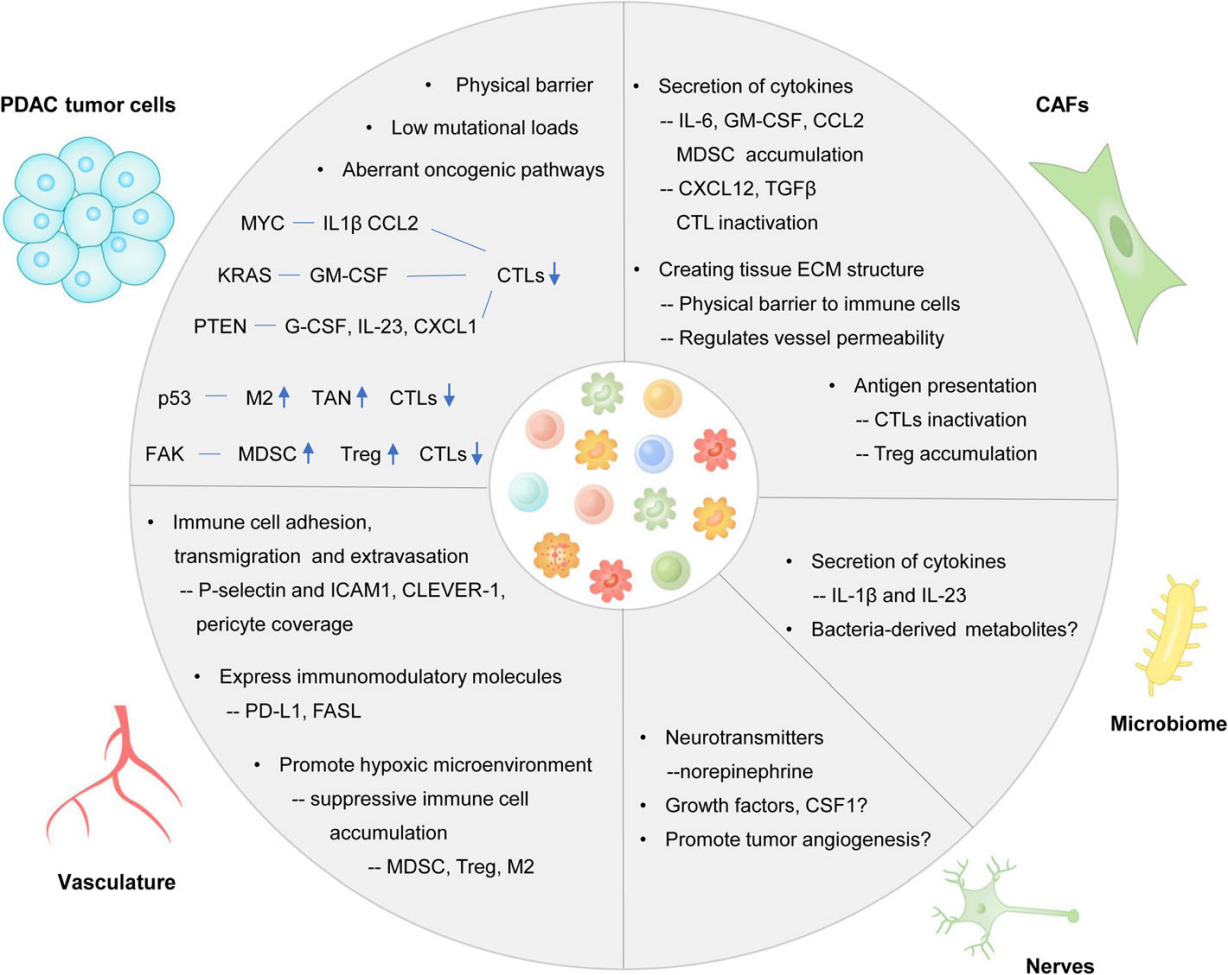
Immune landscape is shaped by tumor tissue components. PDAC tumor cells exert influence on immune infiltration in three ways: establishment of a physical barrier by cellular adhesion and the basal lamina; development of mutational loads; and activation of aberrant oncogenic pathways. Blood vessels control the immune cell composition by affecting immune cell adhesion, transmigration and extravasation. The vascular system also expresses immunomodulatory molecules and provides a hypoxic microenvironment to suppress immune cell accumulation. CAFs mainly modulate the immune microenvironment via cytokine secretion, impacting the ECM structure of the tissue and antigen presentation. The microbiome and nerve fibers can secrete cytokines and neurotransmitters to affect the immune landscape. However, their mechanisms require further exploration

The immune cell composition and functional state vary considerably across tumors, suggesting that the host tissue plays a role in programming the tumor immune landscape. PDAC comprises two distinct components, tumor parenchyma and abundant surrounding stroma. Recent studies have established that dynamic interactions between cancer cells and stromal components modify the immune contexture (Fig. 2).

### Tumor-intrinsic features affect the immune infiltrate

As one type of epithelial-derived carcinoma, PDAC depends on E-cadherin as an intercellular junction protein before undergoing epithelial-to-mesenchymal transition [55]. In addition to this cohesive cellular structure, the basal lamina can separate carcinoma cells from the surrounding tissue. These two structures constitute a physical barrier and limit the entry of some immune cells [56, 57]. MUC1, which is a transmembrane protein produced by epithelial cells, is overexpressed in 90% of PDAC patients and helps cancer cells escape CTLs [12, 58].

In addition to the physical barrier, cancer cell genetic aberrations also have close relationships with the immune cell composition of the TME. Studies have shown that tumors with high mutational burdens, such as non-small-cell lung cancer and melanoma, have higher T cell activity and abundance [59–61]. Although genomic sequencing studies of PDAC have revealed a small set of consistent mutations in most tumors [62], a recently performed genomic analysis identified molecular subtypes of PDAC and found that a small fraction of human PDAC tumors exhibited an immunogenic profile [63]. This evidence indicates additional determinants in cancer cells that contribute to immune contexture in addition to the mutational load.

Balli et al. observed that in sharp contrast to other tumor types, PDAC with high cytolytic immune response levels were linked to genomic copy number alterations rather than the mutational burden. PDAC cases with low cytolytic activity exhibited significantly increased genomic structural variations, such as recurrent amplifications of MYC and NOTCH2 and recurrent deletions and mutations of CDKN2A/B [64]. By studying a mouse model of PDAC, Wormann et al. found that p53 deficiency induced macrophage and neutrophil infiltration while reducing CD8+ T cell levels via JAK2-STAT3 and ROS activation [65]. In addition, a study found that FAK amplification also increased the MDSC, TAM and Treg frequencies and decreased the CTL frequency via STAT3 signaling [66]. A large number of studies have indicated that specific genetic aberrations of cancer cells affect the PDAC immune landscape by orchestrating inflammatory conditions. Inhibition of endogenous MYC led to a significant decrease in infiltrating macrophage and neutrophil frequencies and resulted in tumor regression [67]. Studies in a pancreatic mouse model have demonstrated that MYC amplification stimulates the production of the potent proinflammatory cytokines IL-1β and CCL5, leading to the recruitment of pro-tumoral mast cells in tumor tissue [68, 69].

The effects of genetic determinants on the tumor immune landscape are not limited to only the genes and pathways mentioned above; several other genetic events and downstream immune effects have been described. KRAS is a famous oncogene in PDAC and has been reported to facilitate myeloid cell accumulation in tumors via cytokine release. KRAS-induced secretion of granulocyte-macrophage colony-stimulating factor (GMCSF) results in an influx of CD11b + Gr1+ immunosuppressive cells in PDAC, and ablation of this cytokine impairs immunosuppressive cell accumulation in PDAC tumor tissue, consequently resulting in an increase in the CD8+ T cell frequency [70]. Moreover, loss of both the KRAS and PTEN oncogenes promotes marked activation of NF-κB and its cytokine network, which is accompanied by infiltration of immune cells with known tumor-promoting properties. PTEN/PI3K pathway alteration is a common event in PDAC development, and loss of PTEN results in increased activation of the NF-kB pathway, driving the expression of several immunoregulators, such as G-CSF, IL-23 and CXCL1, by cancer cells [71].

The studies reviewed above suggest that genetic alterations in PDAC cells not only exert an intrinsic effect on the fate of cancer cells but also have a profound influence on the tumor immune landscape. However, most research concentrates on a particular gene and ignores the multitude of genetic and epigenetic alterations that occur simultaneously in tumor progression. In addition, most studies focused on the primary tumor, and events leading to metastasis are largely unaddressed. The interplay between cancer cells and the immune microenvironment will be evaluated in depth with increasingly sophisticated methodologies. Further help is needed to maximize the potential of immunological combination therapies based on the genetic profiles of tumors.

### Stromal components impact immune composition

The tumor mutational burden and intrinsic features of cancer cells can explain immune evasion in PDAC, but they may not explain the whole picture. One of the hallmarks of PDAC is an intensely desmoplastic stroma. Beyond immune cells, the stromal compartment of PDAC includes CAFs; ECM components such as collagen, blood and lymphatic vessels; nerves; and a microbiome, all of which have been shown to affect the antitumor immune response. This section primarily focuses on the strong influence of the stroma on the creation of an immunosuppressive environment in PDAC.

#### Heterogeneous fibroblasts

The abundance of activated fibroblasts and the fibroblast-derived matrix tends to be the most prominent feature of the PDAC microenvironment [72]. The effects of CAFs on cancer progression can be pleiotropically involved in distinct processes, reflecting that CAFs are a population characterized by heterogeneity and plasticity, which may depend on their different origins to a large extent. CAFs originating from the same cellular sources can transdifferentiate into functionally distinct subtypes depending on the context. Heterogeneous fibroblasts shape the architecture of PDAC by creating the tissue ECM structure and secreting cytokines, chemokines and growth factors [73, 74]. In addition to the effects of CAFs on cancer cells, CAFs also impact cancer evolution by programming immune populations, which is dependent on the cellular subtype and complex TME [75].

Given the key role of ECM components in creating the physical barrier in PDAC, CAFs can have a strong impact on restricting access by infiltrating immune cells. Dense collagen networks, which are released from CAFs, represent a physical barrier that can rearrange the T cell distribution and lead to the inhibition of activated T cell migration in dense collagen [76, 77]. However, CAFs mediate ECM remodeling, which can release proinflammatory cytokines and unmask cryptic binding sites, and may promote immune cell adhesion to cancer cells.

Öhlund et al. identified inflammatory cancer-associated fibroblasts (iCAFs) as a subpopulation of CAFs distinct from myofibroblasts in cocultures of murine pancreatic stellate cells (PSCs) and PDAC organoids. iCAFs are located distantly from neoplastic cells and are characterized by intensely elevated expression of cytokines and chemokines such as IL-6 and CXCL1 [78]. High levels of IL-6 production in pancreatic CAFs have been reported to prevent macrophage differentiation and lock recruited monocytes in an immature, suppressive state in a STAT3-dependent manner [79–81]. In addition to interleukins, the cytokines GM-CSF and CCL2 secreted by CAFs mediate an influx of MDSCs into tumor tissue and support metastatic outgrowth [82, 83]. Garg et al. reported that in addition to exerting an influence on the modulation of MDSCs, NF-kB activity in CAFs prevented CTLs from infiltrating PDAC by increasing the expression of CXCL12 [84]. CAFs are also a major source of TGFβ, and recent studies have implicated TGFβ in Th17 cell differentiation, indicating that the CAF immunomodulatory functions have additional complexity [85]. These studies revealed that the secretome heterogeneity of CAFs was implicated in directly regulating antitumor immunity. However, due to CAF secretome heterogeneity and its differential impacts on tumor progression at different states, it is difficult to firmly define the crucial functions of CAF-derived cytokines in regulating immunomodulatory responses. A more precise, functional list of the immunomodulatory cytokines and chemokines produced by CAFs is needed. A fibroblast-specific deletion of crucial cytokines and chemokines in preclinical tumor models may be a promising approach to address this issue.

Recent secretome analyses have reinforced the notion that CAFs modulate immune cell recruitment to the tumor site and immune cell activation by releasing inflammatory molecules, while stromal cells in the lymph node have been shown to induce CD4+ T cell dysfunction through a mechanism involving peptide-MHCII complexes [86]. These observations suggest a potential mechanism by which CAFs exert influence on the immune contexture of tumors. Recently, Elyada et al. applied single-cell RNA sequencing to PDAC tumor tissue samples from six human patients and KPC mice and identified a new CAF subtype named antigen-presenting CAFs (apCAFs). These CAFs could express MHC class II on the cellular membranes and possess the capacity to present antigens to CD4+ T cells. Furthermore, as these apCAFs lacked the costimulatory molecules needed to induce T cell proliferation, CD4+ T cells were hypothesized to be deactivated and to differentiate into Tregs [87]. This study supports the premise that CAFs have the capacity to act as nonprofessional antigen-presenting cells (APCs) and inhibit T cell responses. Some studies on colon tissue and lung cancers have reported that a subset of CAFs expressing PD-L1 and PD-L2 can exert an immunosuppressive effect on T cell activation [88, 89]. In previous studies, we also found PD-L2 expression in the PDAC stroma, but the role and mechanism of PD-L2 in PDAC remain to be further explored [90]. Because PDL1 is difficult to detect in PDAC, it is difficult to investigate immunosuppressive signaling mediated by molecules on the CAF membrane.

Changes in CAF phenotypes during tumor progression exert a heterogeneous and dynamic influence on antitumor immunity. However, these dynamic changes may challenge the dominant view of the immunosuppressive and tumor-promoting roles of CAFs. It has been reported that CAFs may restrain cancer progression as depletion of αSMA+ stromal cells promote an immunosuppressive tumor milieu and exacerbates cancer progression, resulting in diminished survival of PDAC patients [91].

#### Sparse vascular system

For immune cells to exert an effect on cancer cells, they first need to penetrate deep into the tumor through the vascular system [92]. Compared with a normal vascular system, the vascular system in tumor tissue possesses an abnormal structure and facilitates the extravasation of immune cells [93]. Recently, a study demonstrated that the T cell response to an ICB might involve cells that had just recently entered the tumor rather than preexisting tumor-specific T cells [94]. This finding offers strong evidence that the vasculature contributes to regulating immune infiltration and the efficacy of cancer immunotherapies.

PDAC angiogenesis is directly controlled by the proangiogenic factor VEGF, but the continued production of VEGF results in excessive vessel proliferation and rapid but aberrant blood vessel formation [95]. The extensive deposits of fibrotic stroma in PDAC induce elevated interstitial hypertension and vascular compression, leading to a hypoxic microenvironment and excessive VEGF production. The low oxygen levels contribute to a sparse and leaky PDAC vascular system, which is highly specific with a defective basement membrane and abnormal pericyte coverage. These features lead to adjacent endothelial cells (ECs) being loosely attached to one another, which results in leaky tumor blood vessels and subsequently decreases the recruitment of effector immune cells [96]. Hypoxia also increases the accumulation of MDSCs and Tregs within the TME and facilitates the differentiation and polarization of macrophages into the immunosuppressive M2 phenotype [97, 98]. Hypoxia induces high concentrations of the metabolites adenosine and lactate in the TME, resulting in T cell anergy and exhaustion [99]. Excessive VEGF generally inhibits the expression of vascular adhesion molecules such as ICAM-1 and VCAM-1, producing ECs that cannot generate the interactions with T cells necessary for the T cells to cross the endothelial layer and transit into the tumor site [100, 101]. It is not surprising that the sparse immune cell presence in the PDAC stroma is highly resistant to cancer immunotherapies [102].

Furthermore, a study observed that ECs in PDAC expressed relatively high levels of addressins, which could interact with their specific ligands expressed specifically on Tregs. This interaction allowed the selective transmigration of Tregs from the peripheral blood to the tumor tissue and facilitated the immunosuppressive environment [103]. ECs also preferentially attract immunosuppressive Tregs by upregulating the multifunctional endothelial receptor CLEVER-1/stabilin-1 [104]. In vivo studies will be required to further investigate these selective interactions between Tregs and the tumor vasculature mediated by addressins and their respective ligands.

In addition to having effects on immune cell adhesion, transmigration and extravasation into tumor tissue, ECs also shape the tumor immune landscape by expressing immunomodulatory molecules. Recent work has demonstrated that VEGF can enhance the expression of PD-L1 in ECs, thus disabling the cytotoxic function of PD1-positive T cells [105, 106]. A study analyzing tissue microarrays of human cancers showed the existence of FASL in addition to PD-L1 on ECs in ovarian cancer lesions. FASL-expressing ECs assisted immune tolerance by triggering apoptosis in Fas-expressing CD8+ T cells and killing effector T cells [107]. These mechanisms consequently lead to a potent barrier that disables CTL infiltration into the tumor and provide suggestions for further investigations of PDAC.

The features of blood vessels have been widely explored, but the influence of lymphatic vessels on the immune landscape is poorly understood. Lymphatic vessels communicate information and transport immune cells, antigens, and signals from the periphery to the draining lymph node (dLN), implying that lymphatic vessels are required for initiating an immune response against a growing tumor. However, some reports have observed that an increased density of tumor-associated lymphatic vessels correlates with poor patient survival in melanoma and other cancers [108, 109]. The confusing actions of lymphatic vessels in the antitumor immune response and tumor evolution require further investigation.

#### Other stromal factors

Recent reports have generated insights into cancer microbiomes due to revolutionary omics technologies [110], and the potential contribution of the microbiome in pancreatic carcinogenesis has been recognized [111]. Concrete mechanisms are mainly involved in the modulation of the immune microenvironment and antitumor immunity because of the intimate associations of both microbes and cancer with inflammation [112]. Pushalkar et al. showed that bacterial ablation in an orthotopic PDAC mouse model protected against invasive PDAC by reshaping the TME, including reducing MDSC numbers, polarizing macrophages into the M1 phenotype, promoting Th1 differentiation and activating CD8+ T cells. Mechanistically, the PDAC microbiome improved immune surveillance and increased sensitivity to immunotherapy by differentially activating select toll-like receptors (TLR), including TLR2 and TLR5 in monocytic cells [113–115]. Bacterial products may be recognized by TLRs, as has been previously described, or may stimulate the inflammasome-mediated secretion of cytokines, such as the lung microbiome-stimulated IL-1β and IL-23 cytokines produced by myeloid cells, which in turn induces the proliferation and activation of lung-resident γδ T cells [116]. Furthermore, Riquelme et al. demonstrated that the gut microbiome modulates the PDAC tumor microbiome landscape, such as an intra-tumoral microbiome signature (*Pseudoxanthomonas-Streptomyces-Saccharopolyspora-Bacillus clausii*), which is highly predictive of long-term survivorship [117]. That study was the first report that showed microbiota reconstitution in PDAC patients with stool containing the gut microbiome, supporting a causal role for the gut microbiome in shaping the cancer immune environment and PDAC progression. However, the concrete mechanism of how fecal microbiota transplantation induces changes in the tumor microbiome and immune activation in human PDAC patients requires further study. The different taxa of the microbiome coexist in a carefully maintained balance, and affecting one taxon may influence the others. Therefore, more studies are required to investigate the contributions of nonbacterial microbiota-like viruses and fungi to PDAC immune modulation and to identify new specific components of microbiome-immune crosstalk.

Increased innervation and neural hypertrophy are common phenomena in dense pancreatic TME, but the biological signaling of nerves in PDAC is not well understood. Recent reports have highlighted the role of nerve fibers in PDAC evolution, with the density and distortion of the neuronal architecture associated with overall prognosis [118, 119]. Nerve fibers generally elicit cellular effects by releasing neurotransmitters such as catecholamines and acetylcholine, which then bind to α- and β-adrenergic receptors. Immunohistochemical staining analysis in some studies has shown that there is a close correlation between TAMs and nerve density in PDAC tissue [120, 121], indicating a possible paracrine signaling interaction between nerves and macrophages in PDAC tissue. In addition to neurotransmitters, growth factors, such as CSF1, are also released by enteric neurons to communicate with macrophages in healthy tissue [122], suggesting that cytokines are potential mediators of neuro-immune crosstalk in PDAC. More recently, studies have shown that nerve-derived noradrenaline can modulate tumorigenesis by regulating oxidative metabolism in tumor ECs and promoting tumor angiogenesis [123]. As we reviewed in the previous section, blood vessels and ECs are key regulators of immune cell infiltration into the TME. This information reminds us that nerve fibers may influence immune cell infiltration indirectly through impacting vessels in PDAC. A superior understanding and exploration of neuronal signaling and its interactions with immune cells are essential for the development of new therapeutics.

### New therapeutic opportunities

**Fig. 3:**
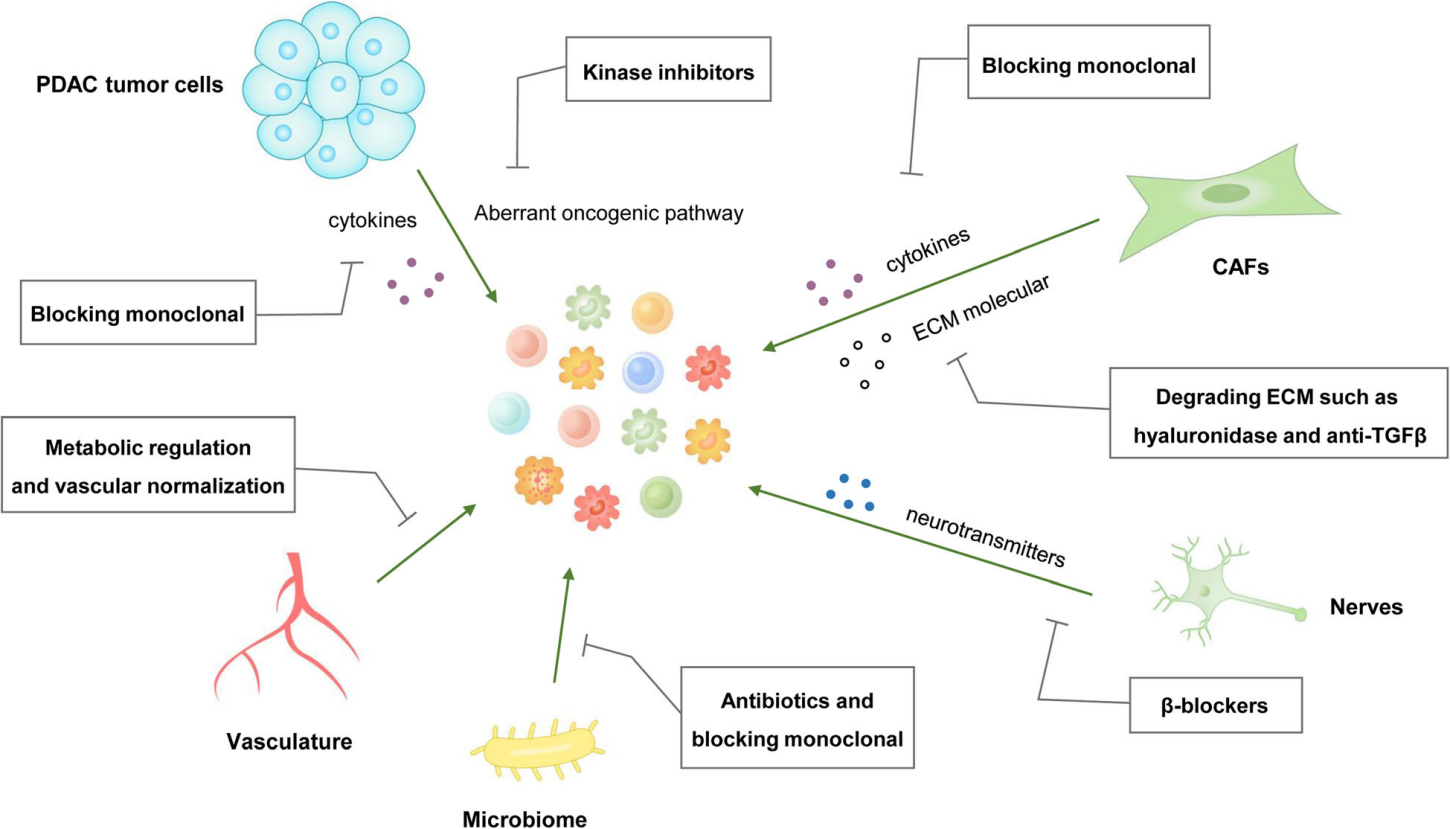
Therapeutic implications of targeting host tissue to activate immune cell infiltration. Implications of therapies that target host tissue, including signaling pathway inhibition, cytokine neutralization, ECM degradation, vascular normalization, antibiotics and β-blockers

The list of approved ICB drugs has grown in recent years, and many cancer therapies have been renewed because of these drugs. However, ICB has not shown significant clinical activity in patients with PDAC because of the low mutational load [124]. As reviewed above, there are many other mechanisms by which host tissue regulates the immune response in PDAC. This suggests that patients resistant to ICB may benefit from tissue-specific modulation strategies. We can induce a favorable immune environment that is sensitive to immunomodulatory drugs by selectively targeting these mechanisms (Fig. 3).

Aberrant signaling pathways in cancer cells lead to the establishment of an immunosuppressive microenvironment in PDAC by recruiting immunosuppressive cells. FAK amplification in pancreatic cancer cells has been identified as an important regulator in the TME, with increases the MDSC, TAM and Treg frequencies and concurrently decreases the CD8+ CTL frequency. These changes remind us that inhibiting FAK amplification in PDAC may induce a favorable immune contexture. Indeed, a study showed that FAK inhibition rendered the previously unresponsive KPC mouse model responsive to T cell immunotherapy and PD-1 antagonists [66]. This study indicates that interference with this cancer cell-intrinsic signaling pathway promotes tumor sensitivity to immunotherapy. Other studies have shown that some targeted drugs that work on cancer cells can indirectly change the immune contexture of tumors by exerting effects on cancer-immune cell crosstalk. Ibrutinib, a drug targeting Bruton tyrosine kinase, can regulate B cell- and macrophage-mediated T cell suppression and can restore T cell-dependent antitumor immune responses to inhibit PDAC growth in PDAC-bearing mice [125]. Insights into the combined effects of targeting cancer cell-intrinsic features will help us expand therapies based on immunomodulatory strategies.

In addition to cancer cell-intrinsic features, the intensely desmoplastic stroma in PDAC exerts strong effects on immune cell infiltration. The immune response is based on the presence of immunomodulatory cells and molecules, such as fibroblasts, ECs in the vascular structure, ECM components and other molecules. The stroma sustains the immunosuppressive environment and affects the immune response in two ways. The first is limiting immune cell influx physically through tight stromal packaging and limited vascularization. The second is reprogramming immune populations via the secretion of chemokines and cytokines [126].

CAFs secreting ECM components cause stromal stiffness and increased hydrostatic pressure. The increased hydrostatic pressure within the PDAC TME is believed to create a barrier to immune cell infiltration. Improving immune cell accessibility to tumor tissue is a good way to increase the efficacy of immunotherapies. Promising results have been obtained with drugs targeting ECM molecules, such as PEGylated human recombinant hyaluronidase (PEGPH20), which enzymatically degrades HA. A phase II trial of PEGPH20 was established for patients with untreated stage IV metastatic PDAC, and the results showed that this drug was well tolerated and might have therapeutic benefits in patients with advanced PDAC, especially in those with high-HA tumors [127]. Furthermore, Caruana et al. engineered chimeric antigen receptor T (CAR-T) cells to express heparanase, which improved their capacity to degrade the ECM and found that T cell infiltration and antitumor activity were promoted [128]. CAFs adopt a secretory phenotype, enabling the production of cytokines and chemokines implicated in regulating antitumor immunity directly. Studies have shown that disrupting IL-6 signaling using anti-IL6R antibodies can improve the chemotherapeutic efficacy in treating PDAC in KPC mice [79]. This finding suggests that neutralizing cytokines from CAFs is a promising way to establish a favorable immune context. However, direct targeted depletion of fibroblasts in PDAC has produced mixed results. Studies have shown that depleting α-SMA+ fibroblasts induces immunosuppression and is related to poor survival in mice and patients [91, 129]. In contrast, in experiments where FAP+ fibroblasts were depleted, improved outcomes were observed in mouse models of PDAC [130, 131]. These divergent conclusions indicate that the distinctions between fibroblast populations are important and that treatments must precisely target specific fibroblast populations in PDAC.

The tumor vasculature assists in establishing the immunosuppressive environment by impacting T cell transmigration and extravasation, by potentially expressing immune inhibitory molecules such as PD-L1 and by enhancing the hypoxic microenvironment. Stromal hydrostatic pressure induces vascular compression within PDAC, and the hypoxic microenvironment and excessive VEGF production lead to a dysfunctional vascular structure, which influences immune cell infiltration [102]. These events suggest that vessel normalization can increase vascular barrier function and tumor perfusion, subsequently facilitating the infiltration and activation of effector immune cells to complement cancer immunotherapies. Jain et al. showed that vascular normalization decreased the interstitial fluid pressure (IFP) within the TME, thereby reducing the restrictions on effector immune cell mobilization and tumoricidal functions [96]. In a preclinical study, Zhao et al. developed an oligonucleotide-based inhibitor (CD5–2) that increased VE-cadherin expression, subsequently normalizing vessel structure and enhancing vessel function. CD5–2 could increase tumor-specific T cell infiltration and spatially redistribute CD8+ T cells within the tumor parenchyma [132]. It is interesting to note that T cells also play an important role in vasculature reprogramming, resulting in immune reprogramming [133]. It is possible that vascular normalization may be a prerequisite to maximizing T cell infiltration and functions in a loop.

Translational research on suppressive cytokines, such as the CXCL family, in PDAC tissue has been of interest for a long time. According to basic research, inhibition of CXCR4 in KPC mice treated with anti-PD-L1 resulted in a modest tumor response (~ 15%) in short-term experiments [134]. Although the related clinical trial NCT02472977 was terminated because of a lack of efficacy in the short-term acute phase, other clinical trials, such as NCT02826486, have not been completed thus far. Both cancer cells and the TME in PDAC have an incredible heterogeneity, and patients with KRAS mutations or abundant FAP+ fibroblasts may have a positive response to this strategy based on basic research. A more precise exploration of this therapeutic strategy is required in a select group of PDAC patients. We hope that other clinical trials will provide promising results in the future.

Although studies on the contribution of the microbiome in PDAC are far from sufficient, antibiotic treatment has shown a preliminary effect. Microbiome depletion leads to a significant increase in IFNγ production by T cells with corresponding decreases in IL-17A and IL-10 production by T cells in PDAC [135], and antibiotics were shown to increase intra-tumoral CD45+ cell infiltration in NOD/SCID mice [136]. Targeting nerve fibers has been proven to be useful in the treatment of PDAC in mouse studies. Renz et al. showed that a β-adrenergic receptor agonist could increase the survival of patients with PDAC and that bilateral adrenalectomy increased the survival of a murine Kras-driven model of PDAC [137]. Neuro-immune crosstalk may be a novel specific component to target, but more investigation is required to prove its contributions to the effects of the abovementioned β-blockers.

Oncolytic viruses (OVs) are currently seen as an emerging alternative therapy for patients with PDAC [138]. OVs can be engineered to express transgenes and replicate in tumor cells to directly induce tumor cell lysis. OVs may be most usefully deployed with ICB as they can be used to modulate the TME by recruiting tumor-infiltrating lymphocytes (TILs), priming immune responses or modifying the vasculature to alter the antitumor immune response [139]. The combination of pelareorep with pembrolizumab and chemotherapy in patients with advanced, previously treated PDAC has been administered in a phase Ib trial. Pelareorep is an oncolytic reovirus that can induce an inflamed T cell-infiltrated (hot) phenotype in PDAC. The safety profile was acceptable, and the efficacy results were encouraging [140]. However, the application of OV–ICB combinations is still in its early stages. More precise studies are required to identify suitable candidate patients and assess the potential for rationally designed OV–ICB combination treatments. The specific tumor contexture, including the barriers for viral entry and the natural tropism of viruses, should be well understood in pretreatment biopsies.

The immune context is programmed by direct and indirect interactions between cellular and molecular components in PDAC tissue. Patients with PDAC are most likely to benefit from a combinatorial but tailored use of strategies that target cancer cells or stromal constituents to prevent immunosuppressive mechanisms and drive effective immune infiltration.

## Conclusions

We conclude that PDAC is characterized by T cell exhaustion and the infiltration of tumor-promoting immune cells, such as M2 macrophages and MDSCs, resulting in poor clinical outcomes. Apart from genetic alterations, PDAC is a type of tumor that has an intensely desmoplastic stroma and a sparse vascular system. Heterogeneous CAFs exert a heterogeneous and dynamic influence on antitumor immunity during tumor progression by remodeling the ECM and secreting cytokines. The sparse vascular system limits immune cell infiltration into tumor tissue and determines the immune landscape by expressing immunomodulatory molecules. Other stromal factors, such as microbiome and nerve fibers, also form a sophisticated interaction network that determines the immune landscape within the TME and has critical roles in the effectiveness of cancer immunotherapies. According to basic research and clinical trials, the effects of therapies that target the stroma, such as ECM degradation, cytokine blockage and vascular normalization, may offer new therapeutic opportunities for PDAC. Deep and dynamic knowledge of the interactions between tumor tissue and the immune response helps with understanding the mechanisms of immune evasion and identifying strategies for combination immunotherapies.

## Data Availability

The materials that support the conclusion of this review have been included within the article.

## Abbreviations

apCAFs: Antigen-presenting CAFs
APCs: Antigen-presenting cells
CAFs: Cancer-associated fibroblasts
CAR-T: Chimeric antigen receptor T
CTL: Cytotoxic T lymphocyte
DCs: Dendritic cells
dLN: Draining lymph node
ECM: Extracellular matrix
ECs: Endothelial cells
GEMM: Genetically engineered mouse model
HA: Hyaluronan
iCAFs: Inflammatory cancer-associated fibroblasts
ICB: Immune checkpoint blockade
IFP: Interstitial fluid pressure
IL-4: Interleukin 4
MDSCs: Myeloid-derived suppressor cells
NO: Nitric oxide
PD-1: Programmed death receptor-1
PDAC: Pancreatic ductal adenocarcinoma
PSCs: Pancreatic stellate cells
ROS: Reactive oxygen species
TAMs: Tumor-associated macrophages
TLR: Toll-like receptors
TME: Tumor microenvironment
Tregs: Regulatory T cells
VEGF: Vascular endothelial growth factor
